# Cognitive Screening of Federal Public Leaders: MoCA Performance, Cognitive Risk, and Predictors of Variability in a Highly Educated Workforce

**DOI:** 10.1111/sjop.70117

**Published:** 2026-05-13

**Authors:** Telesmagno Neves‐Teles, Guilherme da Silva Freitas, Lígia Vechi de Oliveira, Dionatan Pelinson, Cristian Zanon, Rosa Maria Martins de Almeida

**Affiliations:** ^1^ Universidade Federal do Rio Grande Sul (UFRGS) Porto Alegre Brazil

**Keywords:** cognitive reserve, cognitive screening, cognitive vulnerability, episodic memory, intellectual functioning, neurocognitive assessment, public sector leadership

## Abstract

Cognitive screening is essential for assessing mental health and identifying early cognitive vulnerability. This preliminary phase of a preregistered umbrella project (OSF: https://osf.io/8jz6k) implemented a structured cognitive assessment interview with federal public managers, representing an unprecedented initiative in Brazil. The main objective was to characterize the cognitive status of federal public leaders using a multimodal screening protocol and to examine the contributions of intellectual functioning and age to global cognitive performance. Seventy‐eight leaders (M_age_ = 48.44, SD = 8.04) completed a standardized remote cognitive screening session including the Mini‐Mental State Examination, the Brazilian version of the Montreal Cognitive Assessment, and a dyadic short form of the Wechsler Adult Intelligence Scale‐III to estimate intellectual functioning. Brazilian normative data were applied for classification. Associations among age, intellectual functioning, and cognitive performance were examined using bivariate correlations, hierarchical regression, and bootstrap procedures with 5000 resamples. Mini‐Mental State Examination scores were uniformly high (28.12 ± 1.74), reflecting ceiling effects typical of highly educated adults. Although most participants performed within the healthy range on the global Montreal Cognitive Assessment score (25.56 ± 2.70), the Memory Index Score identified a comparatively larger proportion of individuals within normative ranges associated with mild or more pronounced cognitive vulnerability, indicating heightened sensitivity to subtle episodic‐memory changes. Intellectual functioning was a strong positive predictor of cognitive performance, whereas age emerged as a significant negative predictor only after controlling for intellectual functioning, consistent with cognitive‐reserve models. Bootstrap confidence intervals supported the robustness of both predictors. The findings indicate preserved global cognition alongside meaningful heterogeneity in episodic‐memory performance, underscoring the value of memory‐sensitive indices for early cognitive monitoring in high‐responsibility leadership contexts. Subsequent executive‐function assessments from the umbrella project will further refine the identification of domain‐specific cognitive profiles.

## Key Points

- This study provides a multimodal cognitive screening of federal public managers in Brazil.
- Memory‐sensitive indices (MoCA‐MIS) revealed variability not captured by MoCA global cognitive scores.
- Intellectual functioning emerged as a strong predictor of cognitive performance.
- Age effects on cognition were observed only after controlling for intellectual functioning.
- Findings highlight the importance of sensitive cognitive measures in high‐responsibility leadership contexts.

## 1 Introduction

Public‐sector leaders are routinely required to make complex decisions, integrate large volumes of information, and exercise emotional and behavioral regulation under sustained pressure. Such demands depend heavily on executive functions (EFs; Balthazard et al. 2012), which are known to decline subtly with age and to be vulnerable to chronic occupational stress (Diamond 2013; Turnbull et al. 2025). Despite this, the cognitive health status of public leaders remains largely undocumented in empirical research, particularly in middle‐aged adults occupying high‐stakes administrative roles.

As part of the preregistered umbrella study *Executive Functions and Social, Emotional, and Behavioral Skills as Predictors of Transformational Leadership in Federal Public Managers* (OSF registration: https://osf.io/8jz6k), a structured cognitive screening protocol was implemented as an eligibility procedure before the administration of the full EF test battery, socio‐emotional inventories, and leadership measures. This initial phase assessed general cognitive status using the Mini‐Mental State Examination (MMSE), the Montreal Cognitive Assessment (MoCA), and a dyadic short form of the WAIS‐III consisting of the Information and Matrix Reasoning subtests, an approach supported by validated short‐form regression methods for estimating general intellectual functioning (IQ; Denney et al. 2015).

The primary purpose of this screening was to ensure that all participants met the minimal cognitive‐health criteria required for participation in the main study, with an eligibility cutoff of MMSE ≥ 24 (Kochhann et al. 2010). However, the preliminary findings revealed a nontrivial proportion of leaders scoring within the mild cognitive impairment range or, more rarely, within the dementia‐range cutoff based on Brazilian MoCA norms. Additionally, a moderately strong association emerged between global MoCA performance and estimated IQ, consistent with literature suggesting that individuals with high‐cognitive reserve or high intelligence may partially mask early executive or episodic‐memory dysfunction (Barulli and Stern 2013; Kang et al. 2018; Montemurro et al. 2023). Age also showed a negative association with MoCA scores, despite the sample's uniformly high educational attainment.

These findings highlight the importance of systematically documenting cognitive health in leadership populations, both to inform organizational well‐being initiatives and to contextualize subsequent analyses of EF, socio‐emotional skills, and leadership outcomes. Therefore, the present article reports the cognitive screening results as a standalone investigation, aiming to: (a) characterize the cognitive profile of federal public leaders using Brazilian norms; (b) examine associations between MoCA performance, IQ, age, and sex; and (c) evaluate the extent to which MoCA performance is predicted by general intelligence and age in a high‐functioning, highly educated adult sample.

### 1.1 Study Hypotheses

#### 1.1.1 H1. Greater Score Dispersion in Moca Relative to MMSE

Given the well‐documented ceiling effects of the MMSE in highly educated adults, instruments with higher cognitive demand are expected to show greater sensitivity to subtle interindividual variability. Prior studies show that the MMSE performs adequately for moderate impairment but poorly identifies mild deficits, particularly in samples with substantial cognitive reserve (De Boer et al. 2025; Jia et al. 2021; Kang et al. 2018; Montemurro et al. 2023; Stern et al. 2019). Thus, we hypothesize that variability in MoCA total scores will be significantly greater than variability in MMSE scores, as reflected by larger dispersion indices (standard deviation and score range).

#### 1.1.2 H2. Higher Detection of Normative Cognitive Risk Using the MoCA‐MIS

Memory‐specific indices have been shown to detect subtle episodic‐memory vulnerability that may remain undetected by global screening measures, particularly in cognitively preserved and high‐reserve populations. The MoCA‐MIS (Montreal Cognitive Assessment Memory Index Score) is recognized as a highly sensitive marker of delayed episodic‐memory vulnerability, often revealing early memory‐related risk profiles that remain undetected by global cognitive indices, particularly in adults exposed to cumulative cognitive demands, stress, and aging processes (Apolinario et al. 2018; Gonçalves et al. 2025; Julayanont et al. 2013; Kronenberger et al. 2025). We hypothesize that the proportion of participants classified within normative cognitive‐risk ranges will be significantly higher when classification is based on the MoCA‐MIS than when based on the global MoCA score.

#### 1.1.3 H3. Positive Predictive Effect of Estimated IQ on MOCA Performance

Cognitive‐reserve frameworks propose that higher intellectual efficiency contributes to the maintenance of global cognitive performance despite emerging domain‐specific vulnerabilities, particularly among high‐functioning adults with elevated educational attainment (Gu et al. 2025; Kang et al. 2018; Montemurro et al. 2023; Stern et al. 2019). We hypothesize that estimated IQ will positively predict global MoCA performance in hierarchical regression analyses.

#### 1.1.4 H4. Suppression Effect of Age After Controlling for Estimated IQ

In high‐functioning adult samples, age‐related cognitive decline may remain partially masked by cognitive reserve and become detectable only when variance associated with intellectual capacity is statistically controlled (Darby et al. 2017; Elkana et al. 2016; Lee et al. 2025; Ren et al. 2025). From a lifespan developmental perspective, such patterns may reflect adaptive regulatory processes through which individuals maintain effective performance despite emerging functional constraints, as conceptualized in the Selective Optimization with Compensation (SOC) model (Baltes 1997). Accordingly, we hypothesize that age will negatively predict MoCA performance only after controlling for estimated IQ.

#### 1.1.5 H5. Low Prevalence of Normative Cognitive Vulnerability in a High‐Reserve Leadership Sample

Highly educated and professionally active adults are generally expected to show preserved global cognitive performance, with only a minority presenting indicators of normative cognitive vulnerability (Fisher et al. 2017), consistent with evidence of substantial interindividual variability in normative cognitive aging (Castanho et al. 2021). We hypothesize that a relatively small proportion of participants will be classified within normative ranges suggestive of cognitive vulnerability based on MoCA‐derived indices.

## 2 Methods

### 2.1 Participants

A total of 78 federal public leaders participated in the cognitive screening phase of the preregistered umbrella study (OSF: https://osf.io/8jz6k). The sample comprised 44 men and 34 women, with ages ranging from 28 to 72 years (M = 48.44, SD = 8.04). Participants were drawn from the official national directory of high‐level management positions and were required to meet predefined cognitive eligibility criteria before proceeding to subsequent phases of the study.

All participants were neurotypical adults (MMSE ≥ 24), held at least a university degree, and reported no history of neurological or psychiatric disorders. Written informed consent was obtained prior to participation, and all study procedures were approved by the Ethics Committee of the Institute of Psychology, Federal University of Rio Grande do Sul (UFRGS; Protocol no. 7.274.390; CAAE 84134724.5.0000.5334).

### 2.2 Procedure

Screening was conducted remotely, via secure video‐conferencing platforms, following rigorously standardized procedures validated by certified neuropsychologists. Interviewers adhered to a uniform administration protocol to guarantee consistency across participants.

Each cognitive screening session lasted between 45 and 60 min, including standardized instructions, rapport establishment, administration of tests, and scoring checks. This screening phase was conducted between July and December 2025, prior to the collection of the full executive‐function battery, socio‐emotional measures, and leadership assessments that constitute the main components of the umbrella study.

### 2.3 Instruments

#### 2.3.1 Mini‐Mental State Examination (MMSE)

The MMSE was administered according to the Brazilian norms of Brucki et al. (2003). An eligibility cutoff of ≥ 24 was adopted for participation in subsequent phases of the project, following the recommendations of Kochhann et al. (2010) for individuals with higher educational attainment (sensitivity = 87%, specificity = 82%).

#### 2.3.2 Montreal Cognitive Assessment—Brazilian Version (MoCA)

The MoCA was administered following Memória et al. (2013) and interpreted according to the Brazilian normative stratification proposed by Apolinario et al. (2018). Both the total MoCA score and the MoCA‐MIS were calculated. The MoCA‐MIS offers a relatively precise index of delayed episodic memory, in accordance with findings from the original MoCA validation study (Nasreddine et al. 2005) and with subsequent refinements emphasizing its sensitivity to episodic memory processes (Julayanont et al. 2013). Cognitive‐status classification followed Brazilian *Z*‐score norms: Healthy (*Z* > −1.0); Mild Cognitive Impairment (MCI) range (−2.0 < *Z* ≤ −1.0); and Major Neurocognitive Disorder (MNCD) range (*Z* ≤ −2.0). For participants under 50 years, the normative cutoffs for ages 50–59 were applied as the most conservative available criterion.

Distribution of global MoCA scores did not meet strict normality (*Shapiro–Wilk p* < 0.001), but Skewness = −0.504 and Kurtosis = −0.632 were within the acceptable −1 to +1 range (Field 2005), supporting the use of parametric analyses with caution.

#### 2.3.3 WAIS‐III Dyadic Short Form (Information + Matrix Reasoning)

General intellectual ability was estimated using the WAIS‐III Information (I) and Matrix Reasoning (MR) subtests. This dyadic short form follows validated regression‐based methods described in the Brazilian WAIS‐III normative updates (Wechsler 2020) and in international references on WAIS‐IV short forms (Denney et al. 2015).

The Information + Matrix Reasoning dyad is supported for predicting general intellectual functioning, minimizing administration time in remote protocols, and providing stable reliability in neurologically healthy adults. Age‐adjusted scaled scores from these subtests were converted to *z*‐scores and averaged to derive an estimated IQ score used in all statistical analyses. The sex variable was treated as a categorical factor (1 = female, 2 = male) and was not standardized.

### 2.4 Data Analysis

Statistical analyses were conducted using Jamovi, JASP, and R. Descriptive statistics were computed for all cognitive measures, and participants were classified according to Brazilian MoCA normative criteria. Differences in cognitive‐status classification based on the global MoCA score and the MoCA‐MIS were examined using McNemar's chi‐square test for paired proportions.

Associations among global MoCA performance, MoCA‐MIS, estimated IQ, age, and sex were examined using Pearson correlation coefficients. Hierarchical linear regression analyses were conducted to evaluate predictors of cognitive performance. For both global MoCA scores and MoCA‐MIS, three‐step models were specified: Model 1 included estimated IQ; Model 2 additionally included age; and Model 3 included sex as a control variable to examine potential confounding effects.

In addition to conventional regression estimates, bootstrap procedures with 5000 resamples were applied to assess the robustness of regression coefficients, using bias‐corrected and accelerated (BCa) 95% confidence intervals. Model evaluation included changes in explained variance (ΔR^2^), *F*‐change statistics, standardized coefficients, and verification of key regression assumptions, including residual normality, homoscedasticity, absence of multicollinearity, and independence of errors. Detailed diagnostic outputs and supplementary analyses are provided in the Supporting Information.

Effect sizes were interpreted using conventional benchmarks (Cohen 1988, 1992), including small, moderate, and large effects for correlation coefficients (*r* = 0.10, 0.30, 0.50), Cohen's *d* (0.20, 0.50, 0.80), and coefficients of determination (*R* ^2^ = 0.02, 0.13, 0.26). Statistical significance was set at α = 0.05.

## 3 Results

### 3.1 Descriptive Statistics

The sample comprised 78 federal public managers (34 women and 44 men). Participants were middle‐aged adults, with ages ranging from 28 to 72 years (M = 48.44, SD = 8.04); no significant age difference was observed between men (M = 48.64, SD = 7.90) and women (M = 48.18, SD = 8.32; *p* = 0.804). Skewness and kurtosis values indicated acceptable distributions for both groups; Shapiro–Wilk tests also supported normal age distribution in the sample (W = 0.977, *p* = 0.159). Full descriptive statistics are presented in Table 1.

**TABLE 1:** Descriptives and cognitive results.

	Total (*N* = 78)	Male (*n* = 44)	Female (*n* = 34)	*p* (*t*‐test)
Age (SD)	48.44 (8.04)	48.64 (7.91)	48.18 (8.32)	0.804
MMSE (SD)	28.12 (1.74)	28.07 (1.81)	28.18 (1.68)	0.788
MoCA (SD)	25.56 (2.70)	26.02 (2.82)	24.97 (2.46)	0.088
MoCA‐MIS (SD)	3.253 (1.57)	3.32 (1.60)	3.44 (1.46)	0.584
IQ (SD)	116.60 (7.92)	118.16 (8.20)	114.59 (7.17)	0.048

Abbreviations: IQ, intellectual quotient; MMSE, mini‐mental state examination; MoCA, Montreal cognitive assessment; MOCA‐MIS, Montreal Cognitive Assessment Memory Index Score.

Global cognitive functioning measured by the MMSE was uniformly high (M = 28.12, SD = 1.74), with no significant differences between men and women (*p* = 0.788). Performance on the MoCA followed a similar pattern, with slightly higher mean scores among men but no statistically significant sex differences (*p* = 0.088), and MoCA‐MIS performance was virtually identical across groups (*p* = 0.584). Despite the high mean scores, both the MMSE and the MoCA deviated significantly from normality, as indicated by Shapiro–Wilk tests (MMSE: *W* = 0.87, *p* < 0.001; MoCA: *W* = 0.94, *p* = 0.002), consistent with ceiling effects in this highly educated sample.

Estimated IQ scores derived from the WAIS‐III Information + Matrix Reasoning dyad were normally distributed (*W* = 0.976, *p* = 0.156). The relatively high mean of 116.60 (SD = 7.92; range = 96–136) reflects the elevated educational and professional profile of the leadership cohort. A statistically significant difference in estimated IQ was observed between men and women (*p* = 0.048), with a small‐to‐moderate effect size (Cohen's *d* = −0.46, 95% CI [−0.91, −0.01]).

### 3.2 MoCA Normative Classification

Normative classification according to Brazilian standards (Apolinario et al. 2018) was conducted using both the global MoCA score and the MoCA‐MIS. Results for each index are shown in Tables 2 and 3, respectively.

**TABLE 2:** Cognitive status based on the global MoCA score.

Cognitive status	*N* (F)	Age (SD)	MoCA score (SD)
Healthy	64 (26)	48.7 (7.77)	26.5 (1.93)
MCI	12 (6)	48.4 (8.05)	21.6 (1.31)
MNCD	2 (2)	41.5 (19.09)	20.0 (1.41)

Abbreviations: F, Female; MCI, mild cognitive impairment; MNCD, major neurocognitive disorder; MoCA, Montreal cognitive assessment.

**TABLE 3:** Cognitive status based on the delayed‐recall index (MoCA‐MIS).

Cognitive status	*N* (F)	Age (SD)	MoCA‐MIS (SD)
Healthy	44 (21)	48.32 (8.08)	4.43 (0.66)
MCI	16 (6)	47.43 (9.41)	2.75 (0.45)
MNCD	18 (7)	48.89 (7.39)	1.17 (0.86)

Abbreviations: F, Female; MCI, mild cognitive impairment; MNCD, major neurocognitive disorder; MoCA‐MIS, Montreal Cognitive Assessment Memory Index Score.

As shown in Table 2, most participants demonstrated performance within the healthy range (*n* = 64), with a mean MoCA score of 26.5 (SD = 1.93). Twelve participants scored within the mild neurocognitive disorder (MCI) range, and two participants scored within the range consistent with potential major neurocognitive disorder (MNCD). Although these classifications do not constitute diagnostic determinations, they indicate early signs of cognitive risk in a small proportion of the sample.

A different pattern emerged when classification was based exclusively using the MoCA‐MIS. As shown in Table 3, fewer participants were classified as healthy (*n* = 44), whereas a larger proportion fell within the MCI (*n* = 16) and MNCD (*n* = 18) normative ranges. This distribution suggests that the delayed episodic‐memory component of the MoCA may be more sensitive to subtle cognitive vulnerability than the global score in highly educated adult samples.

To formally examine differences in classification patterns between indices, McNemar's test was conducted using a dichotomized cognitive‐risk variable (healthy vs. at risk). Results revealed a significant asymmetry in classifications, *χ* ^2^(1) = 15.38, *p* < 0.001, indicating that substantially more participants were classified as cognitively at risk when screening was based on the MoCA‐MIS compared with the global MoCA score. Specifically, 23 participants classified as healthy according to the global MoCA were categorized within normative risk ranges using the MoCA‐MIS, whereas only three participants showed the opposite pattern.

Taken together, these findings indicate that although global MoCA performance suggested preserved cognitive functioning in most participants, the memory‐specific index identified a markedly larger subgroup presenting normative indicators of cognitive vulnerability. These results highlight the added value of memory‐sensitive screening indices in cognitively demanding occupational contexts.

### 3.3 Bivariate Associations

Associations among age, estimated IQ, and cognitive screening measures are presented in Table 4. Given deviations from normality in cognitive screening scores, most associations were examined using Spearman's rank‐order correlations (*ρ*), whereas the association between age and estimated IQ, both normally distributed variables, was examined using Pearson's correlation coefficient (*r*).

**TABLE 4:** Associations among age, sex, estimated IQ, and cognitive screening measures.

	Age	Sex	IQ	MoCA	MoCA‐MIS	MMSE
Age	—					
Sex	*ρ* = −0.111	—				
IQ	*r* = 0.178	*ρ* = 0.283*	—			
MoCA	*ρ* = −0.166	*ρ* = 0.238*	*ρ* = 0.495***	—		
MoCA‐MIS	*ρ* = −0.201	*ρ* = 0.028	*ρ* = 0.221	*ρ* = 0.703***	—	
MMSE	*ρ* = −0.138	*ρ* = 0.008	*ρ* = 0.239*	*ρ* = 0.202	*ρ* = 0.240*	—

*Note:* *p < 0.05, ***p < 0.001.

Abbreviations: IQ, intellectual quotient; MMSE, Mini‐Mental State Examination; MoCA, Montreal cognitive assessment; MOCA‐MIS, Montreal Cognitive Assessment Memory Index Score.

Global MoCA performance showed a moderate positive correlation with estimated IQ (*ρ* = 0.495, *p* < 0.001). MMSE scores were also positively correlated with IQ (*ρ* = 0.239, *p* = 0.035), although this association should be interpreted cautiously given the restricted range of MMSE scores in this highly educated sample. MoCA‐MIS performance was not significantly correlated with estimated IQ (*ρ* = 0.221, *p* = 0.053) or age (*ρ* = −0.201, *p* = 0.078), indicating that memory‐specific screening performance was not reliably associated with these variables at the bivariate level.

Age was not significantly correlated with global MoCA performance at the bivariate level (*ρ* = −0.166, *p* = 0.147), nor with estimated IQ (*r* = 0.178, *p* = 0.138). However, when controlling for estimated IQ, age showed a significant negative partial association with MoCA performance (partial *ρ* = −0.291, *p* = 0.010), suggesting that shared variance with intellectual functioning suppressed the observable association between age and global cognitive screening scores.

### 3.4 Hierarchical Regression Analysis

#### 3.4.1 Predictors of Global Cognitive Performance (MoCA Total Score)

Hierarchical linear regression analyses were conducted to examine the contribution of estimated IQ, age, and sex to global MoCA performance.

In Model 1, estimated IQ was entered as the sole predictor and significantly explained variance in MoCA scores, *R* = 0.452, *R* ^2^ = 0.204, adjusted *R* ^2^ = 0.194, *F*(1, 76) = 19.53, *p* < 0.001. Estimated IQ showed a moderate positive association with MoCA performance (β = 0.45, *p* < 0.001).

In Model 2, age was added to the model, resulting in a significant improvement in model fit, Δ*R* ^2^ = 0.066, *F*_change_(1, 75) = 6.83, *p* = 0.011. The full model explained 27.1% of the variance in MoCA scores (*R* = 0.52, *R* ^2^ = 0.271, adjusted *R* ^2^ = 0.251). When controlling for estimated IQ, age emerged as a significant negative predictor of MoCA performance (β = −0.26, *p* = 0.011), whereas estimated IQ remained a strong positive predictor (β = 0.50, *p* < 0.001).

In Model 3, sex was entered as an additional predictor to evaluate potential confounding effects suggested by preliminary group comparisons. The inclusion of sex did not significantly improve model fit (Δ*R* ^2^ = 0.00, *p* > 0.05), and sex did not emerge as a significant predictor of MoCA performance. Importantly, the magnitude and statistical significance of the effects associated with estimated IQ and age remained stable, indicating robustness after controlling for sex.

To evaluate the robustness of these effects, bootstrap analyses with 5000 resamples were conducted using bias‐corrected and accelerated confidence intervals. Bootstrap estimates confirmed a significant positive effect of estimated IQ (*B* = 0.16, 95% BCa CI [0.09, 0.24], *p* < 0.001) and a significant negative effect of age (*B* = −0.09, 95% BCa CI [−0.16, −0.02], *p* = 0.008), supporting the stability of the parameter estimates. Bootstrap estimates for sex were nonsignificant (*p* = 0.331), with confidence intervals including zero.

Diagnostic statistics indicated that key regression assumptions were met. Residual distributions were approximately normal, collinearity indices were low (VIFs close to 1.0), and Durbin–Watson statistics were near 2.0, suggesting independence of residuals. Detailed diagnostic outputs for all hierarchical regression models are provided in the Supporting Information.

#### 3.4.2 Predictors of Episodic‐Memory Performance (MoCA Memory Index Score)

Hierarchical linear regression analyses were conducted to examine the contribution of estimated IQ, age, and sex to episodic‐memory performance as indexed by the MoCA Memory Index Score (MoCA‐MIS).

In Model 1, estimated IQ was entered as the sole predictor and did not significantly explain variance in MoCA‐MIS scores, *R* = 0.166, *R* ^2^ = 0.027, adjusted *R* ^2^ = 0.015, *F*(1, 76) = 2.15, *p* = 0.147. Estimated IQ showed a small and nonsignificant association with episodic‐memory performance (β = 0.17).

In Model 2, age was added to the model, resulting in a significant improvement in model fit, Δ*R* ^2^ = 0.058, *F*_change_(1, 75) = 4.73, *p* = 0.033. The full model explained 8.5% of the variance in MoCA‐MIS performance (*R* = 0.292, *R* ^2^ = 0.085, adjusted *R* ^2^ = 0.061). When controlling for estimated IQ, age emerged as a significant negative predictor of episodic‐memory performance (β = −0.24, *p* = 0.033), whereas estimated IQ showed a small and nonsignificant positive association (β = 0.21, *p* = 0.066).

In Model 3, sex was entered as an additional predictor to evaluate potential confounding effects. The inclusion of sex did not significantly improve model fit, Δ*R* ^2^ = 0.011, *F*_change_(1, 74) = 0.91, *p* = 0.342. In the fully adjusted model, episodic‐memory performance was significantly predicted by both estimated IQ (β = 0.23, *p* = 0.046) and age (β = −0.25, *p* = 0.032), whereas sex did not emerge as a significant predictor (β = −0.11, *p* = 0.342). The final model explained 9.6% of the variance in MoCA‐MIS performance (*R* = 0.310, *R* ^2^ = 0.096, adjusted *R* ^2^ = 0.060).

Bootstrap analyses with 5000 resamples using bias‐corrected and accelerated confidence intervals confirmed the robustness of both predictors in the fully adjusted model. Age showed a significant negative effect on episodic‐memory performance (*B* = −0.03, 95% BCa CI [−0.06, −0.01], *p* = 0.022), whereas estimated IQ showed a significant positive effect (*B* = 0.03, 95% BCa CI [0.01, 0.06], *p* = 0.036). Bootstrap estimates for sex were nonsignificant (*p* = 0.332), with confidence intervals including zero.

Diagnostic statistics similarly indicated no violations of major regression assumptions. Residuals were approximately normally distributed, variance inflation factors were low, and Durbin–Watson statistics were close to 2.0 across models. Full diagnostic information, including residual plots and collinearity statistics, is available in the Supporting Information.

### 3.5 Overall Summary of Results

Across analyses, participants demonstrated generally high levels of global cognitive performance, accompanied by meaningful interindividual variability, particularly on MoCA‐derived indices. Normative classification based on Brazilian standards indicated that the global MoCA score identified relatively few individuals within normative cognitive‐risk ranges, whereas classification based on the MoCA‐MIS revealed a substantially larger subgroup presenting indicators of episodic‐memory vulnerability. This asymmetry was statistically supported by McNemar's test, indicating that memory‐sensitive screening indices may provide incremental sensitivity for detecting subtle cognitive variability in highly educated adult samples.

Bivariate analyses showed a moderate positive association between estimated intellectual functioning and global MoCA performance, whereas age was not significantly associated with MoCA scores at the zero‐order level. However, after accounting for shared variance with intellectual functioning, age emerged as a significant negative predictor of global cognitive performance, consistent with a suppression pattern. Hierarchical regression models further indicated that estimated IQ was the strongest predictor of global MoCA performance, while sex did not significantly contribute to model fit.

For episodic‐memory performance indexed by the MoCA‐MIS, hierarchical models revealed a different pattern. Age emerged as a consistent negative predictor, whereas estimated IQ showed a smaller but significant positive association in the fully adjusted model. Together, these findings suggest that cognitive functioning in this highly educated leadership cohort reflects the joint influence of intellectual resources and age‐related processes, and that delayed episodic‐memory indices may reveal subtle vulnerabilities not fully captured by global cognitive screening measures.

## 4 Discussion

The present study investigated the cognitive status of federal public leaders using a comprehensive remote screening protocol that included the MMSE, the MoCA, and the MoCA Memory Index Score (MoCA‐MIS), and an estimate of general intellectual ability. This investigation provides one of the first systematic assessments of cognitive health in a high‐responsibility leadership cohort in Brazil. Overall, the results revealed preserved global cognition, with meaningful variability emerging in episodic‐memory performance and in age‐ and IQ‐adjusted outcomes. In particular, normative classification and hierarchical regression analyses revealed differential sensitivity between global and memory‐specific screening measures. In the sections that follow, these findings are interpreted in relation to the study hypotheses (H1–H5) and relevant lifespan and cognitive‐reserve frameworks.

### 4.1 Evaluation of the Hypotheses

#### 4.1.1 H1—Greater Score Dispersion in MoCA Relative to MMSE

Consistent with extensive literature, the MMSE scores in this highly educated sample displayed ceiling effects, thereby limiting sensitivity to subtle deficits (Bertolucci et al. 1994; Brucki et al. 2003; Kochhann et al. 2010; Memória et al. 2013). In contrast, the MoCA exhibited broader score variability, confirming its superior capacity to detect mild cognitive changes in cognitively preserved adults (Bergeron et al. 2017; Cesar et al. 2019; Nasreddine et al. 2005; Siqueira et al. 2019).

Importantly, this pattern is consistent with more recent evidence indicating that the MoCA outperforms the MMSE in high‐functioning and high‐cognitive‐reserve populations, where global performance often remains within normal limits despite emerging domain‐specific vulnerabilities (De Boer et al. 2025; Jia et al. 2021; Kang et al. 2018). Together, these findings reinforce the notion that instruments with greater cognitive demands and reduced ceiling effects are essential for detecting early and subtle interindividual variability in highly educated samples. Thus, H1 was supported.

#### 4.1.2 H2—Higher Detection of Normative Cognitive Risk Using the MoCA‐MIS

An important divergence emerged between classifications based on global MoCA scores and the MoCA‐MIS scores. Whereas the global MoCA identified a relatively small proportion of individuals within normative ranges suggestive of MCI or MNCD, the MoCA‐MIS classified a substantially larger subset as exhibiting memory‐related vulnerability. This classification asymmetry was statistically supported by paired comparison analyses, indicating that substantially more participants were identified as cognitively at risk when classification was based on the MoCA‐MIS. This pattern reinforces the MoCA‐MIS as a particularly sensitive marker of delayed episodic‐memory functioning, a cognitive domain consistently shown to reflect early risk even among individuals with high intellectual functioning and preserved global cognition (Apolinario et al. 2018; Julayanont et al. 2013; Kronenberger et al. 2025).

However, reduced delayed‐recall performance should not be interpreted exclusively as indicative of prodromal neurodegenerative processes. Evidence indicates that memory‐specific fluctuations may also arise from stress‐ and mood‐related cognitive interference, which are common in middle‐aged and older adults and tend to disproportionately affect retrieval‐dependent processes (Castanho et al. 2021; Stawski et al. 2006). Such effects may be further amplified in high‐demand professional contexts, where sustained cognitive load places additional strain on attentional control, working memory, and episodic‐memory retrieval mechanisms (Fisher et al. 2017). Taken together, these findings indicate that the MoCA‐MIS captures a broader spectrum of normative memory vulnerability, thereby providing strong support for H2.

#### 4.1.3 H3—Positive Predictive Effect of Estimated IQ on MoCA Performance

Estimated IQ emerged as the strongest positive predictor of global MoCA performance in this cohort, consistent with cognitive reserve frameworks proposing that intellectual enrichment and premorbid cognitive capacity buffer against the expression of subtle cognitive decline in global screening performance (Barulli and Stern 2013). This association aligns with accumulating evidence indicating that individuals with higher cognitive reserve can sustain normative global performance even in the presence of early inefficiencies in cognitive processing or memory retrieval (Gu et al. 2025; Montemurro et al. 2023).

In the present highly educated leadership sample, this dynamic likely contributed to the overall preservation of global MoCA scores, despite the emergence of domain‐specific vulnerabilities revealed by more sensitive memory‐specific indices. These findings underscore the methodological importance of incorporating estimates of intellectual ability when interpreting cognitive screening outcomes in high‐functioning populations. Accordingly, H3 was supported.

#### 4.1.4 H4—Suppression Effect of Age After Controlling for Estimated IQ

Age showed minimal association with MoCA scores at the bivariate level but emerged as a significant negative predictor when estimated IQ was statistically controlled. This suppression pattern suggests that shared variance between age and intellectual capacity partially masked age‐related effects, making them detectable only when individual differences in cognitive reserve were taken into account. Such findings are consistent with previous evidence indicating that higher cognitive reserve may delay the behavioral manifestation of age‐related cognitive decline, particularly in high‐functioning and highly educated adults (Darby et al. 2017; Elkana et al. 2016; Lee et al. 2025; Ren et al. 2025).

From a lifespan developmental standpoint, these results may also reflect adaptive regulatory dynamics through which individuals maintain effective performance in cognitively demanding roles despite emerging domain‐specific vulnerabilities. Within the Selective Optimization with Compensation (SOC) framework, extensions of lifespan models to organizational contexts suggest that sustained occupational functioning depends on the strategic allocation of cognitive resources and the use of compensatory mechanisms that allow individuals to maintain goal attainment under conditions of constrained reserve capacity (Baltes and Dickson 2001; Moghimi et al. 2019). Rather than indicating the absence of decline, the observed pattern suggests that age‐related cognitive changes may remain functionally buffered by preserved intellectual resources and compensatory performance regulation strategies. Accordingly, H4 was supported.

#### 4.1.5 H5—A Larger‐Than‐Expected Subset Displayed Memory‐Related Normative Risk Indicators

Although we initially hypothesized that only a small subset of participants would show normative indicators of mild cognitive vulnerability (H5), the MoCA‐MIS revealed a considerably larger proportion within MCI‐ or MNCD‐range scores than anticipated. This pattern was statistically supported by the significant asymmetry observed in classification analyses comparing global MoCA and MoCA‐MIS outcomes. It likely reflects the heightened sensitivity of the MoCA‐MIS to subtle episodic‐memory fluctuations, which may emerge even in high‐functioning adults exposed to sustained cognitive load and elevated professional demands. Such fluctuations may also be influenced by stress‐related cognitive interference and attentional fatigue, processes commonly observed in demanding occupational environments (Castanho et al. 2021; Fisher et al. 2017).

Importantly, the lack of support for H5 does not undermine the theoretical rationale of limited cognitive risk in high‐reserve populations; rather, it suggests that memory‐specific indices may detect a broader spectrum of normative vulnerability than initially anticipated when global cognitive performance remains preserved. From an applied perspective, this finding constitutes a relevant alert for mental health policies targeting public leaders. Even in the absence of diagnostic implications, the identification of a comparatively elevated prevalence of episodic‐memory vulnerability underscores the need for preventive organizational strategies aimed at monitoring cognitive well‐being, mitigating occupational stress, and supporting sustained cognitive performance in leadership roles.

Thus, H5 was not supported, indicating that episodic‐memory vulnerability may be more prevalent among public leaders than originally expected when assessed using highly sensitive memory‐specific measures such as the MoCA‐MIS.

### 4.2 Theoretical and Practical Implications

The present findings reinforce the conceptual relevance of cognitive reserve as a critical framework for interpreting cognitive performance in high‐functioning adults. Leadership roles entail sustained attention, rapid information integration, complex decision‐making, and continuous self‐regulation, capacities supported by executive and memory systems that are particularly susceptible to the cumulative effects of aging, stress, and chronic cognitive load. In this context, the results underscore that traditional cognitive screening tools, such as the MMSE, may be insufficient to capture emerging vulnerabilities in highly educated and professionally active populations. By contrast, memory‐sensitive indices such as the MoCA‐MIS appear better suited to reveal subtle, domain‐specific variability that remains functionally buffered at the global level.

From a lifespan developmental perspective, these findings may also be interpreted within the framework of Selective Optimization with Compensation (SOC), which conceptualizes adaptive functioning across adulthood as the dynamic regulation of goals and resources through processes of selection, optimization, and compensation (Baltes 1997). Extensions of this model to organizational contexts suggest that effective performance in cognitively demanding roles depends on the strategic allocation of cognitive effort, the refinement of preserved competencies, and the adoption of compensatory mechanisms that allow individuals to maintain goal attainment despite emerging functional constraints (Baltes and Dickson 2001; Moghimi et al. 2019). In leadership settings, such mechanisms may include reliance on accumulated expertise, structured decision routines, delegation strategies, and the use of external cognitive supports.

From an applied perspective, the identification of episodic‐memory vulnerability in a comparatively larger‐than‐anticipated subset of public leaders constitutes a relevant alert for mental health and organizational strategies within public administration. Even in the absence of diagnostic implications, these patterns suggest that subtle cognitive fluctuations may be more prevalent among leaders than traditionally assumed. This finding highlights the importance of integrating cognitive monitoring into organizational well‐being and leadership development programs, not as a diagnostic instrument, but as a preventive and supportive resource. Such initiatives may contribute to early identification of cognitive strain, inform targeted stress‐mitigation strategies, and support the maintenance of cognitive efficiency in roles characterized by sustained decision‐making demands, high information‐processing load, and adaptive leadership performance.

### 4.3 Strengths and Limitations

This study presents several methodological strengths. First, it examines a highly selective and understudied population (federal public leaders) within a preregistered umbrella study. The use of standardized remote assessments conducted by trained interviewers ensured procedural consistency and strong ecological validity in a real‐world occupational setting.

A second strength is the adoption of a multimodal cognitive screening approach that included the MMSE, the global MoCA score, the MoCA‐MIS, and an estimated IQ index. This integrated strategy enabled a more sensitive characterization of cognitive functioning than is typically reported in occupational or leadership research. Moreover, the use of Brazilian age‐ and education‐adjusted MoCA norms (Apolinario et al. 2018), together with bootstrap‐validated regression models, further strengthened the robustness and interpretability of the findings.

Despite these advantages, some limitations must be acknowledged. The cross‐sectional design precludes inferences regarding longitudinal cognitive change. In addition, although the remote assessment protocol was rigorously standardized, remote administration may have influenced performance on tasks relying heavily on memory or attentional control, particularly in the presence of environmental distractions.

Furthermore, the reliance on screening instruments rather than a comprehensive neuropsychological battery means that the classifications reported here reflect normative risk indicators rather than diagnostic determinations. Accordingly, the regression analyses should be interpreted as hypothesis‐guided but partly exploratory attempts to model variability in screening outcomes rather than as confirmatory tests of clinical prediction. The exclusive inclusion of highly educated public leaders, consistent with the aims of the umbrella study, also limits the generalizability of the findings to other educational or occupational contexts.

Finally, while the MoCA‐MIS enhances sensitivity to subtle episodic‐memory changes, it does not capture the full spectrum of executive‐function variability. To address this limitation, the umbrella study incorporates a comprehensive executive‐function battery assessing attention, working memory, inhibitory control, and cognitive flexibility. These complementary measures will enable a more precise identification of domain‐specific cognitive alterations that may remain undetected by global screening tools alone and will allow future longitudinal and multivariate analyses to clarify the functional significance of the present screening findings.

## 5 Conclusion

The present study provides a comprehensive cognitive profile of federal public leaders using a multimodal screening protocol combining global and memory‐focused MoCA indices, MMSE classification, and an estimate of intellectual functioning. Although global cognition was largely preserved, the MoCA‐MIS revealed meaningful heterogeneity in delayed episodic‐memory performance, suggesting that subtle cognitive fluctuations may be more prevalent in this population than previously assumed. Notably, these patterns emerged within a highly educated, high‐functioning cohort, underscoring the role of cognitive reserve mechanisms in shaping variability in screening outcomes.

The identification of a comparatively larger‐than‐anticipated proportion of leaders within normative memory‐risk ranges on the MoCA‐MIS represents an important alert for cognitive health strategies in public administration. While not indicative of clinical impairment, these findings highlight the value of sensitive, domain‐specific screening measures capable of detecting early episodic‐memory vulnerability in individuals exposed to sustained cognitive load, complex decision‐making demands, and occupational stress. Integrating such measures into organizational well‐being initiatives may support early identification of cognitive strain and contribute to the long‐term maintenance of cognitive efficiency in leadership roles.

Finally, these results reinforce the relevance of multimodal cognitive screening approaches and support the next phases of the preregistered umbrella study, which will incorporate detailed assessments of executive functions, socio‐emotional skills, and leadership outcomes. Longitudinal and multivariate investigations will be essential to clarify the functional significance and temporal stability of the episodic‐memory patterns observed. Overall, the present findings advance understanding of cognitive functioning in public leadership and support evidence‐based strategies for promoting cognitive health in contexts of sustained responsibility and high‐cognitive demand.

## Author Contributions

**Telesmagno Neves‐Teles:** conceptualization, methodology, investigation, data curation, formal analysis, writing – original draft, project administration. **Guilherme da Silva Freitas:** investigation, methodology. **Lígia Vechi de Oliveira:** investigation; methodology. **Dionatan Pelinson:** investigation; methodology. **Cristian Zanon:** validation, supervision, writing – review and editing. **Rosa Maria Martins de Almeida:** validation, supervision, writing – review and editing.

## Funding

The authors have nothing to report.

## Ethics Statement

All study procedures were approved by the Ethics Committee of the Institute of Psychology, Universidade Federal do Rio Grande do Sul (UFRGS; Protocol no. 7.274.390; CAAE 84134724.5.0000.5334).

## Consent

Written informed consent was obtained prior to participation.

## Conflicts of Interest

The authors declare no conflicts of interest.

## Supporting information


**Data S1:** Dataset.

**File S1:** Classification asymmetry analysis.

**File S2:** Hierarchical regression models for global MoCA scores.

**File S3:** Hierarchical regression models for MoCA‐MIS scores.

**File S4:** Correlation matrix and bivariate normality diagnostics.

## Data Availability

The data supporting the findings of this study are available as online Supporting Information accompanying this article, including the dataset and Supporting Information [Link], [Link], [Link], [Link].
