# Supplementary material for: Cognitive Screening of Federal Public Leaders: MoCA Performance, Cognitive Risk, and Predictors of Variability in a Highly Educated Workforce
Source: Scand J Psychol. 2026 May 13;67(5):1359–69. doi: 10.1111/sjop.70117 (PMC13546102; doi:10.1111/sjop.70117)

Hier. Regr.: MoCA-MIS ~ IQ, Age, and Sex

Model Summary - MoCA-MIS_z

										Durbin-Watson		
Model	R	R^2	Adjusted R^2	RMSE	R^2 Change	F Change	df1	df2	p	Autocorrelation	Statistic	p
M_0	0.166	0.027	0.015	0.993	0.027	2.145	1	76	.147	-0.157	2.273	.235
M_1	0.292	0.085	0.061	0.969	0.058	4.728	1	75	.033	-0.108	2.163	.498
M_2	0.310	0.096	0.060	0.970	0.011	0.914	1	74	.342	-0.129	2.201	.400

Note. M_0 includes IQ_total_score

Note. M_1 includes IQ_total_score, Age

Note. M_2 includes IQ_total_score, Age, Sex

ANOVA

Model		Sum of Squares	df	Mean Square	F	p
M_0	Regression	2.113	1	2.113	2.145	.147
	Residual	74.89	76	0.985		
	Total	77.00	77			
M_1	Regression	6.555	2	3.277	3.489	.036
	Residual	70.45	75	0.939		
	Total	77.00	77			
M_2	Regression	7.414	3	2.471	2.628	.056
	Residual	69.59	74	0.940		
	Total	77.00	77			

Note. M_0 includes IQ_total_score

Note. M_1 includes IQ_total_score, Age

Note. M_2 includes IQ_total_score, Age, Sex

Coefficients

Model		Unstandardized	Standard Error	Standardized	t	p	95% CI Lower	Upper	Collinearity Statistics Tolerance	VIF
M_0	(Intercept)	-2.439	1.669		-1.461	.148	-5.763	0.885		
	IQ_total_score	0.021	0.014	0.166	1.464	.147	-0.008	0.049	1.000	1.000
M_1	(Intercept)	-1.608	1.674		-0.961	.340	-4.942	1.727		
	IQ_total_score	0.026	0.014	0.209	1.863	.066	-0.002	0.055	0.968	1.033
	Age	-0.030	0.014	-0.244	-2.174	.033	-0.058	-0.003	0.968	1.033
M_2	(Intercept)	-1.623	1.675		-0.969	.336	-4.960	1.714		
	IQ_total_score	0.030	0.015	0.234	2.029	.046	5.240×10^{-4}	0.058	0.920	1.087
	Age	-0.031	0.014	-0.245	-2.185	.032	-0.058	-0.003	0.968	1.033
	Sex	-0.217	0.227	-0.108	-0.956	.342	-0.670	0.236	0.949	1.053

Bootstrap Coefficients

Model		Unstandardized	Bias	Standard Error	p*	95% CI* Lower	Upper
M_0	(Intercept)	-2.442	-0.103	1.649	.118	-5.843	0.656
	IQ_total_score	0.021	8.200×10^{-4}	0.014	.124	-0.006	0.049
M_1	(Intercept)	-1.635	-0.078	1.648	.313	-4.943	1.540
	IQ_total_score	0.026	6.139×10^{-4}	0.014	.065	-0.001	0.055
	Age	-0.030	-4.762×10^{-5}	0.013	.016	-0.058	-0.005
M_2	(Intercept)	-1.711	-0.084	1.693	.363	-4.777	1.869
	IQ_total_score	0.030	8.215×10^{-4}	0.015	.045	3.515×10^{-4}	0.058
	Age	-0.030	-1.372×10^{-4}	0.014	.022	-0.059	-0.005
	Sex	-0.223	-0.007	0.213	.311	-0.637	0.212

Note. Bootstrapping based on 5000 replicates.

Note. Coefficient estimate is based on the median of the bootstrap distribution.

* Bias corrected accelerated.

Descriptives

	N	Mean	SD	SE
MoCA-MIS_z	78	-3.981×10^{-17}	1.000	0.113
IQ_total_score	78	116.6	7.921	0.897
Age	78	48.44	8.040	0.910
Sex	78	1.564	0.499	0.057

Part And Partial Correlations

Model		Partial	Part
M_0	IQ_total_score	0.166	0.166
M_1	IQ_total_score	0.210	0.206
	Age	-0.244	-0.240
M_2	IQ_total_score	0.230	0.224
	Age	-0.246	-0.241
	Sex	-0.110	-0.106

Collinearity Diagnostics

				Variance Proportions			
Model	Dimension	Eigenvalue	Condition Index	(Intercept)	IQ_total_score	Age	Sex
M_0	1	1.998	1.000	0.001	0.001		
	2	0.002	29.67	0.999	0.999		
M_1	1	2.981	1.000	0	0	0.003	
	2	0.017	13.16	0.04	0.044	0.996	
	3	0.002	36.25	0.96	0.956	0.001	
M_2	1	3.911	1.000	0	0	0.002	0.005
	2	0.070	7.465	0.004	0.003	0.052	0.922
	3	0.016	15.43	0.05	0.048	0.945	0.057
	4	0.002	41.84	0.946	0.948	0.001	0.016

Influential Cases

Case Number	Std. Residual	MoCA-MIS_z	Predicted Value	Residual	Cook's Distance
.	.	.	.	.	.

Note. No influential cases found.

Residuals Statistics

	Minimum	Maximum	Mean	SD	N
Predicted Value	-0.850	0.652	-1.941×10^{-15}	0.310	78
Residual	-2.404	1.521	3.981×10^{-17}	0.951	78
Std. Predicted Value	-2.740	2.102	-2.177×10^{-17}	1.000	78
Std. Residual	-2.521	1.622	0.003	1.006	78

Standardized Residuals Histogram

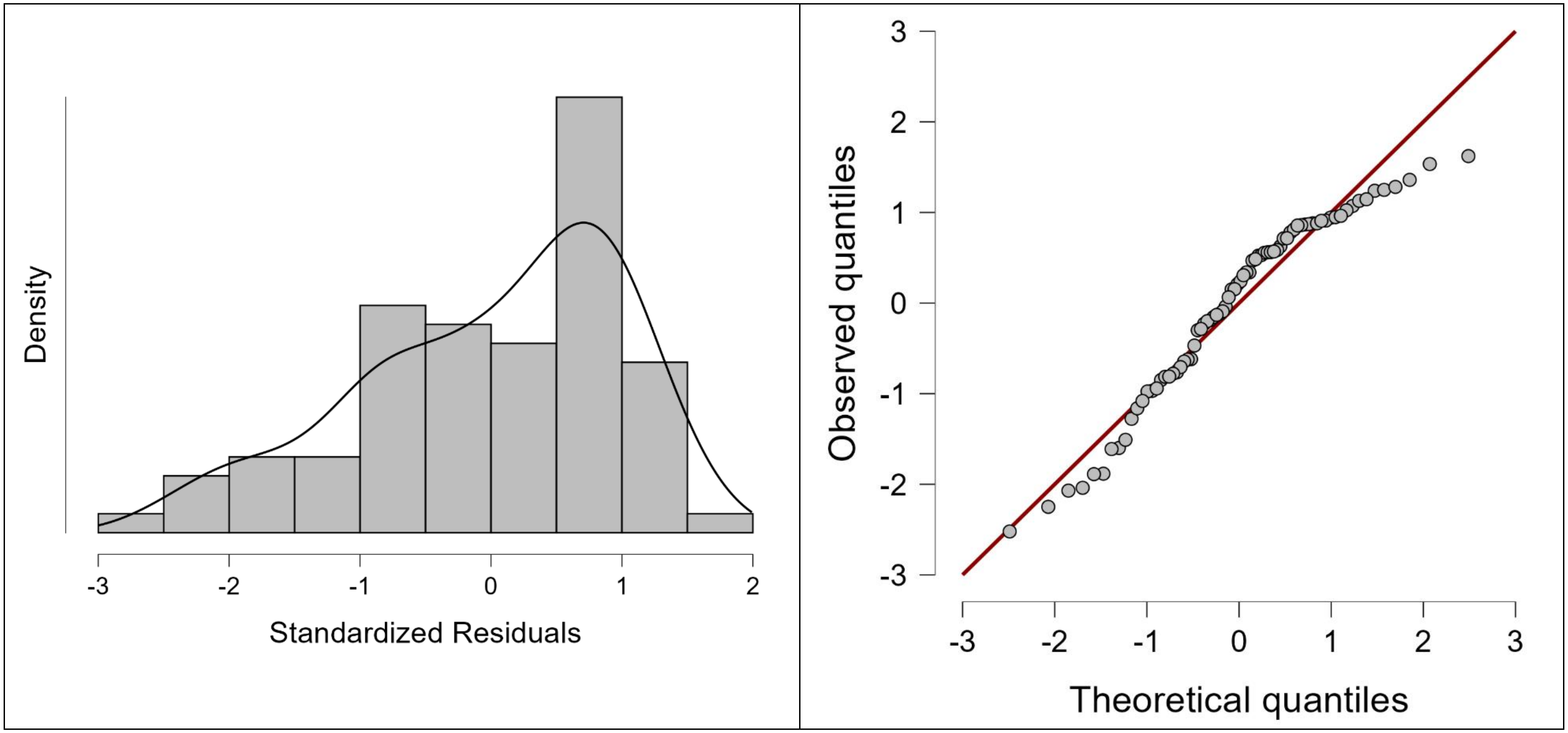

Marginal effect of IQ_total_score on MoCA-MIS_z

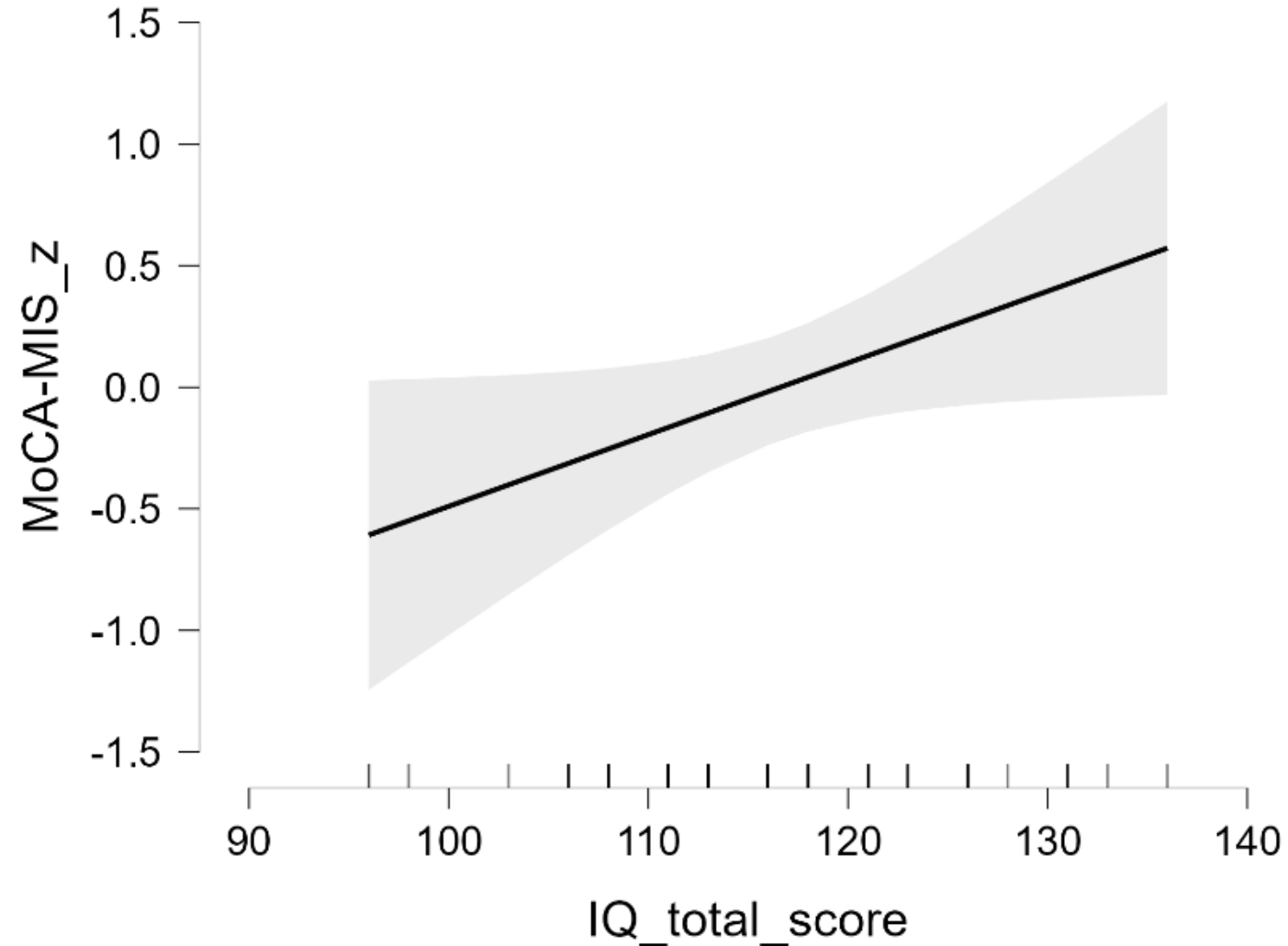

Marginal effect of Sex on MoCA-MIS_z

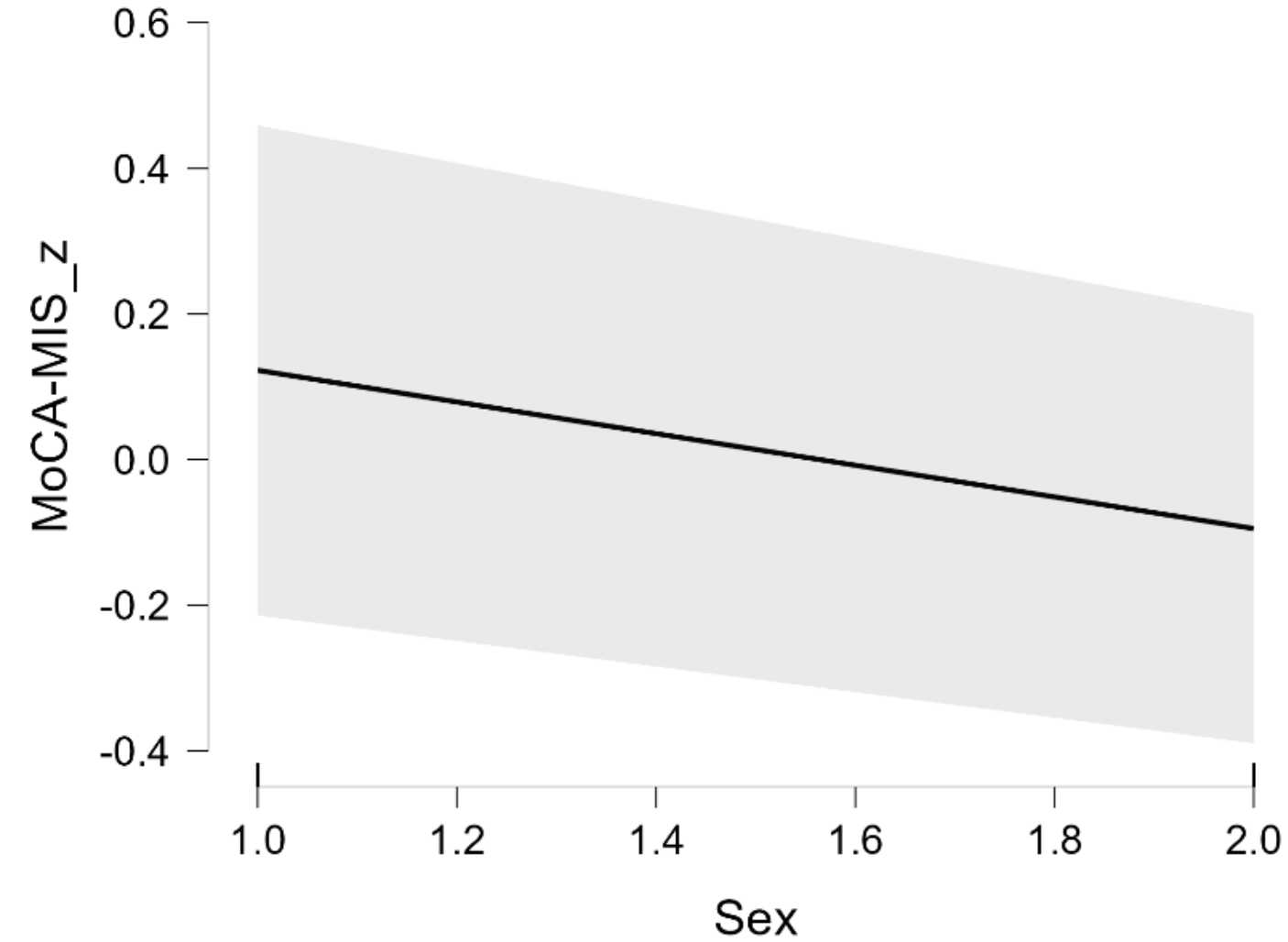

Marginal effect of Age on MoCA-MIS_z

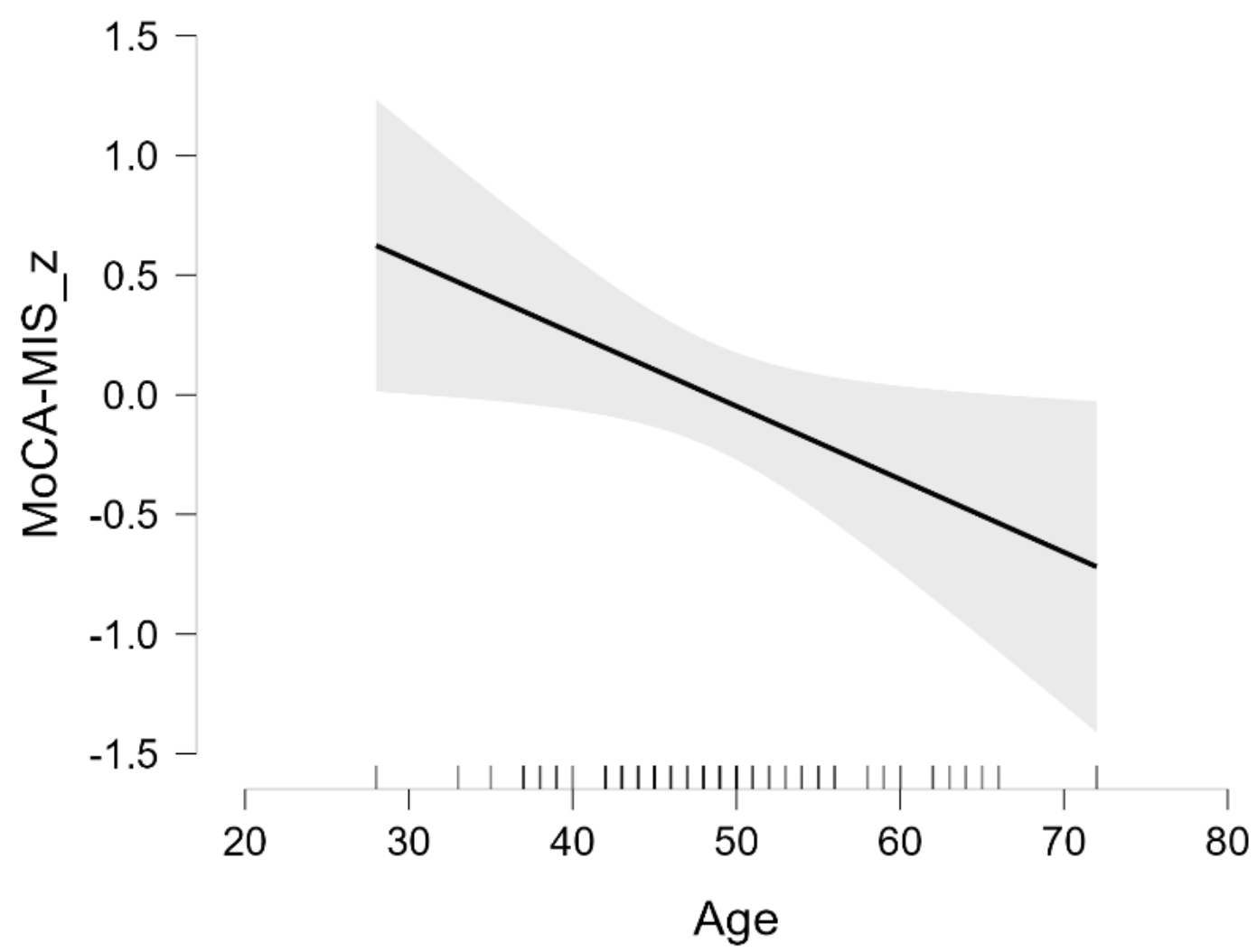

Supplement: Supplementary file 1 [file SJOP-67-1359-s001.pdf]
