# Supplementary material for: Cognitive Screening of Federal Public Leaders: MoCA Performance, Cognitive Risk, and Predictors of Variability in a Highly Educated Workforce
Source: Scand J Psychol. 2026 May 13;67(5):1359–69. doi: 10.1111/sjop.70117 (PMC13546102; doi:10.1111/sjop.70117)
Supplement: Supplementary file 2 [file SJOP-67-1359-s002.pdf]

Supplementary File S4 — Correlation matrix and bivariate normality diagnostics

Correlation Table

			Pearson						Spearman				
			n	r	Lower 95% CI	Upper 95% CI	Effect size (Fisher's z)	SE Effect size	rho	Lower 95% CI	Upper 95% CI	Effect size (Fisher's z)	SE Effect size
moca_total_score_z	-	MoCA-MIS_z	78	0.705***	0.572	0.802	0.877	0.115	0.703***	0.569	0.800	0.873	0.122
moca_total_score_z	-	IQ_total_score_z	78	0.452***	0.255	0.613	0.487	0.115	0.495***	0.306	0.646	0.543	0.119
moca_total_score_z	-	Age	78	-0.173	-0.381	0.051	-0.175	0.115	-0.166	-0.374	0.059	-0.167	0.116
moca_total_score_z	-	Sex	78	0.195	-0.029	0.400	0.197	0.115	0.233*	0.011	0.433	0.237	0.117
MoCA-MIS_z	-	IQ_total_score_z	78	0.166	-0.059	0.374	0.167	0.115	0.221	-0.001	0.423	0.225	0.117
MoCA-MIS_z	-	Age	78	-0.207	-0.410	0.016	-0.210	0.115	-0.201	-0.405	0.023	-0.204	0.117
MoCA-MIS_z	-	Sex	78	-0.063	-0.281	0.162	-0.063	0.115	-0.057	-0.276	0.168	-0.057	0.115
IQ_total_score_z	-	Age	78	0.178	-0.046	0.385	0.180	0.115	0.170	-0.055	0.378	0.171	0.116
IQ_total_score_z	-	Sex	78	0.225*	0.003	0.426	0.229	0.115	0.216	-0.007	0.419	0.220	0.117
Age	-	Sex	78	0.029	-0.195	0.249	0.029	0.115	0.052	-0.172	0.272	0.052	0.115

Note. Pearson correlations are reported for comparability with regression analyses; Spearman coefficients are additionally provided due to deviations from bivariate normality.
* p < .05, ** p < .01, *** p < .001

Assumption checks

Shapiro-Wilk Test for Bivariate Normality

			Shapiro-Wilk	p
moca_total_score_z	-	MoCA-MIS_z	0.959	.002
moca_total_score_z	-	IQ_total_score_z	0.969	.021
moca_total_score_z	-	Age	0.969	.022
moca_total_score_z	-	Sex	0.844	< .001
MoCA-MIS_z	-	IQ_total_score_z	0.947	< .001
MoCA-MIS_z	-	Age	0.957	.001
MoCA-MIS_z	-	Sex	0.779	< .001
IQ_total_score_z	-	Age	0.983	.395
IQ_total_score_z	-	Sex	0.872	< .001
Age	-	Sex	0.816	< .001
