# Supplementary material for: Cognitive Screening of Federal Public Leaders: MoCA Performance, Cognitive Risk, and Predictors of Variability in a Highly Educated Workforce
Source: Scand J Psychol. 2026 May 13;67(5):1359–69. doi: 10.1111/sjop.70117 (PMC13546102; doi:10.1111/sjop.70117)
Supplement: Supplementary file 3 [file SJOP-67-1359-s003.pdf]

Supplementary File S1 — Classification asymmetry analysis

Contingency Tables

	risk_MIS		
**risk_global**	**1**	**0**	**Total**
1	11	3	14
0	23	41	64
Total	34	44	78

McNemar Test

	Value	df	p
**χ^2**	15.38	1	<.001
**χ^2 continuity correction**	13.88	1	<.001
**N**	78		
