# Supplementary material for: Cognitive Screening of Federal Public Leaders: MoCA Performance, Cognitive Risk, and Predictors of Variability in a Highly Educated Workforce
Source: Scand J Psychol. 2026 May 13;67(5):1359–69. doi: 10.1111/sjop.70117 (PMC13546102; doi:10.1111/sjop.70117)

Hier. Regr.: MoCA Global ~ IQ, Age, and Sex

Model Summary - moca_total_score

										Durbin-Watson		
Model	R	R^2	Adjusted R^2	RMSE	R^2 Change	F Change	df1	df2	p	Autocorrelation	Statistic	p
M_0	0.452	0.204	0.194	2.424	0.204	19.53	1	76	< .001	-0.020	2.026	.931
M_1	0.520	0.271	0.251	2.336	0.066	6.825	1	75	.011	-0.042	2.062	.818
M_2	0.528	0.279	0.250	2.338	0.008	0.872	1	74	.354	-0.026	2.029	.962

Note. M_0 includes IQ_total_score

Note. M_1 includes IQ_total_score, Age

Note. M_2 includes IQ_total_score, Age, Sex

ANOVA

Model		Sum of Squares	df	Mean Square	F	p
M_0	Regression	114.7	1	114.7	19.53	< .001
	Residual	446.4	76	5.874		
	Total	561.2	77			
M_1	Regression	152.0	2	75.99	13.93	< .001
	Residual	409.2	75	5.456		
	Total	561.2	77			
M_2	Regression	156.7	3	52.25	9.560	< .001
	Residual	404.4	74	5.465		
	Total	561.2	77			

Note. M_0 includes IQ_total_score

Note. M_1 includes IQ_total_score, Age

Note. M_2 includes IQ_total_score, Age, Sex

Coefficients

Model		Unstandardized	Standard Error	Standardized	t	p	95% CI Lower	95% CI Upper	Collinearity Statistics Tolerance	Collinearity Statistics VIF
M_0	(Intercept)	7.594	4.075		1.863	.066	-0.523	15.71		
	IQ_total_score	0.154	0.035	0.452	4.420	< .001	0.085	0.224	1.000	1.000
M_1	(Intercept)	10.00	4.034		2.479	.015	1.964	18.04		
	IQ_total_score	0.170	0.034	0.499	4.977	< .001	0.102	0.238	0.968	1.033
	Age	-0.088	0.034	-0.262	-2.613	.011	-0.155	-0.021	0.968	1.033
M_2	(Intercept)	10.04	4.038		2.485	.015	1.990	18.08		
	IQ_total_score	0.163	0.035	0.477	4.639	< .001	0.093	0.233	0.920	1.087
	Age	-0.088	0.034	-0.261	-2.599	.011	-0.155	-0.020	0.968	1.033
	Sex	0.512	0.548	0.095	0.934	.354	-0.580	1.603	0.949	1.053

Bootstrap Coefficients

Model		Unstandardized	Bias	Standard Error	p*	95% CI* Lower	95% CI* Upper
M_0	(Intercept)	7.434	-0.221	4.320	.061	-0.432	16.65
	IQ_total_score	0.156	0.002	0.036	< .001	0.078	0.220
M_1	(Intercept)	9.862	-0.124	4.695	.020	1.487	19.94
	IQ_total_score	0.170	0.002	0.038	< .001	0.093	0.244
	Age	-0.088	-0.001	0.035	.007	-0.163	-0.023
M_2	(Intercept)	9.897	-0.088	4.631	.016	1.666	19.87
	IQ_total_score	0.163	0.002	0.039	< .001	0.088	0.240
	Age	-0.088	-0.002	0.035	.006	-0.160	-0.025
	Sex	0.506	-0.016	0.534	.340	-0.539	1.556

Note. Bootstrapping based on 5000 replicates.

Note. Coefficient estimate is based on the median of the bootstrap distribution.

* Bias corrected accelerated.

Descriptives

	N	Mean	SD	SE
moca_total_score	78	25.56	2.700	0.306
IQ_total_score	78	116.6	7.921	0.897
Age	78	48.44	8.040	0.910
Sex	78	1.564	0.499	0.057

Part And Partial Correlations

Model		Partial	Part
M_0	IQ_total_score	0.452	0.452
M_1	IQ_total_score	0.498	0.491
	Age	-0.289	-0.258
M_2	IQ_total_score	0.475	0.458
	Age	-0.289	-0.256
	Sex	0.108	0.092

Collinearity Diagnostics

				Variance Proportions			
Model	Dimension	Eigenvalue	Condition Index	(Intercept)	IQ_total_score	Age	Sex
M_0	1	1.998	1.000	0.001	0.001		
	2	0.002	29.67	0.999	0.999		
M_1	1	2.981	1.000	0	0	0.003	
	2	0.017	13.16	0.04	0.044	0.996	
	3	0.002	36.25	0.96	0.956	0.001	
M_2	1	3.911	1.000	0	0	0.002	0.005
	2	0.070	7.465	0.004	0.003	0.052	0.922
	3	0.016	15.43	0.05	0.048	0.945	0.057
	4	0.002	41.84	0.946	0.948	0.001	0.016

Influential Cases

Case Number	Std. Residual	moca_total_score	Predicted Value	Residual	Cook's Distance
.	.	.	.	.	.

Note. No influential cases found.

Residuals Statistics

	Minimum	Maximum	Mean	SD	N
Predicted Value	21.81	28.46	25.56	1.427	78
Residual	-5.152	5.450	-6.725×10^{-17}	2.292	78
Std. Predicted Value	-2.629	2.027	5.216×10^{-16}	1.000	78
Std. Residual	-2.236	2.443	0.002	1.008	78

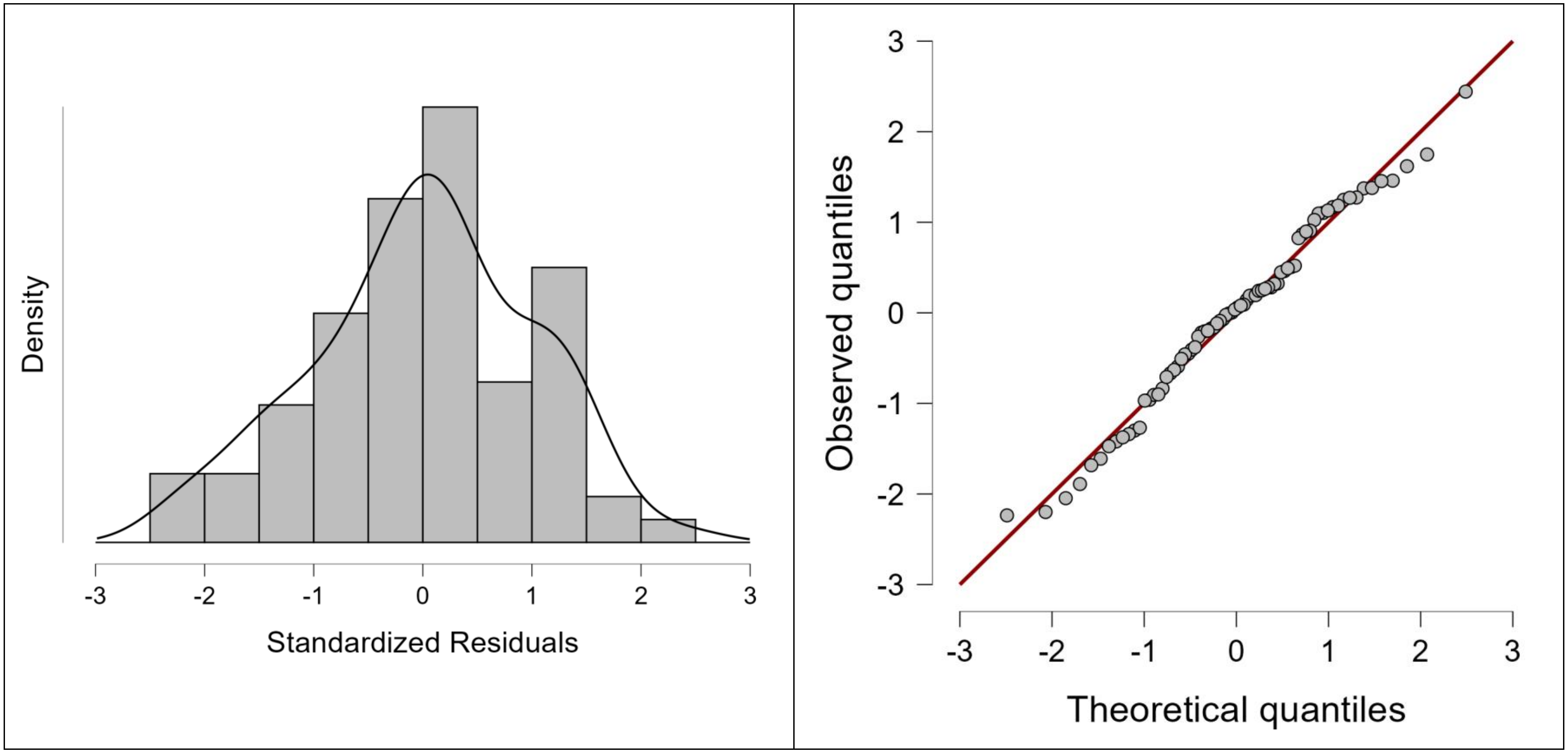

Marginal effect of IQ_total_score on moca_total_score

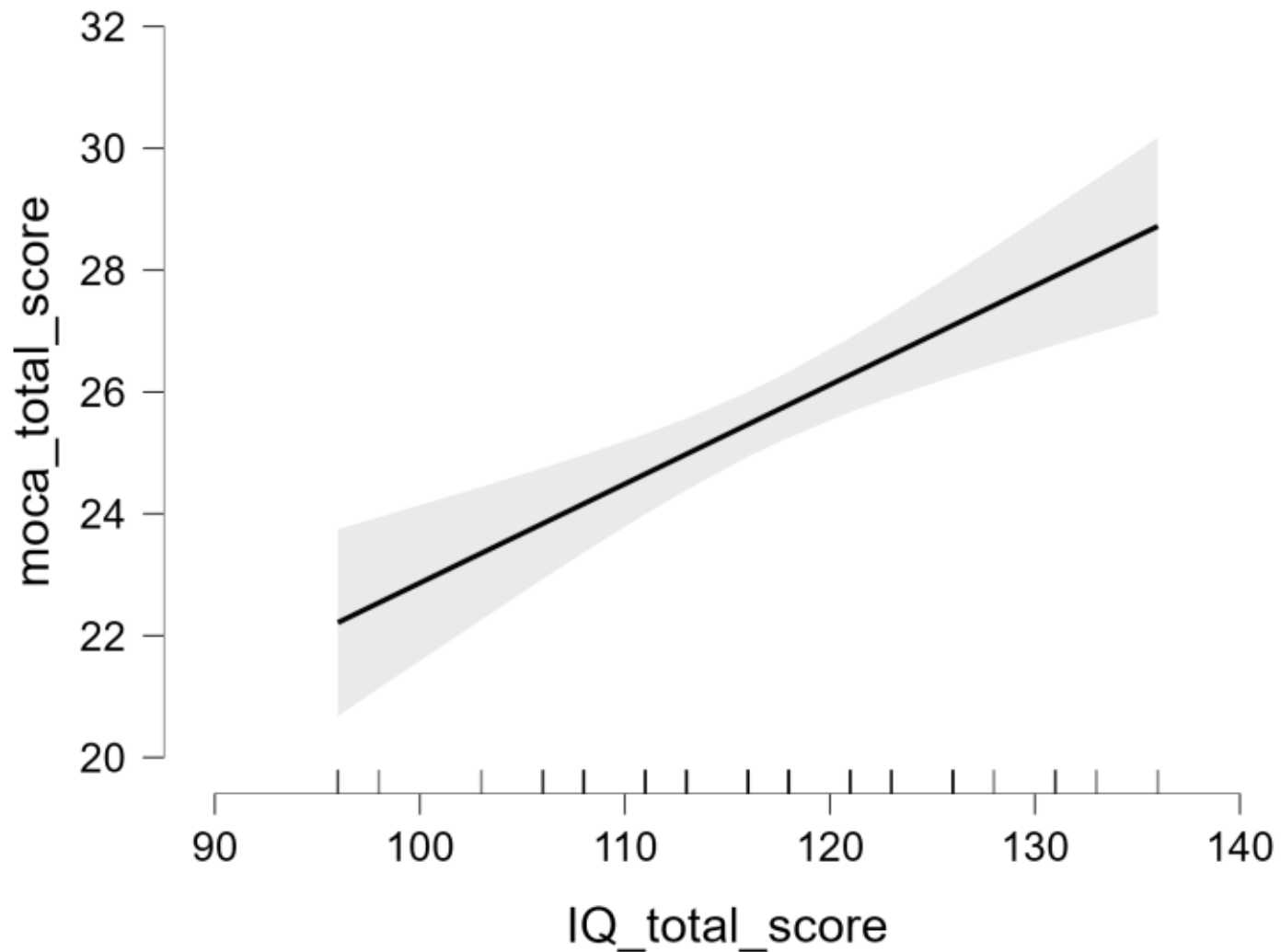

Marginal effect of Sex on moca_total_score

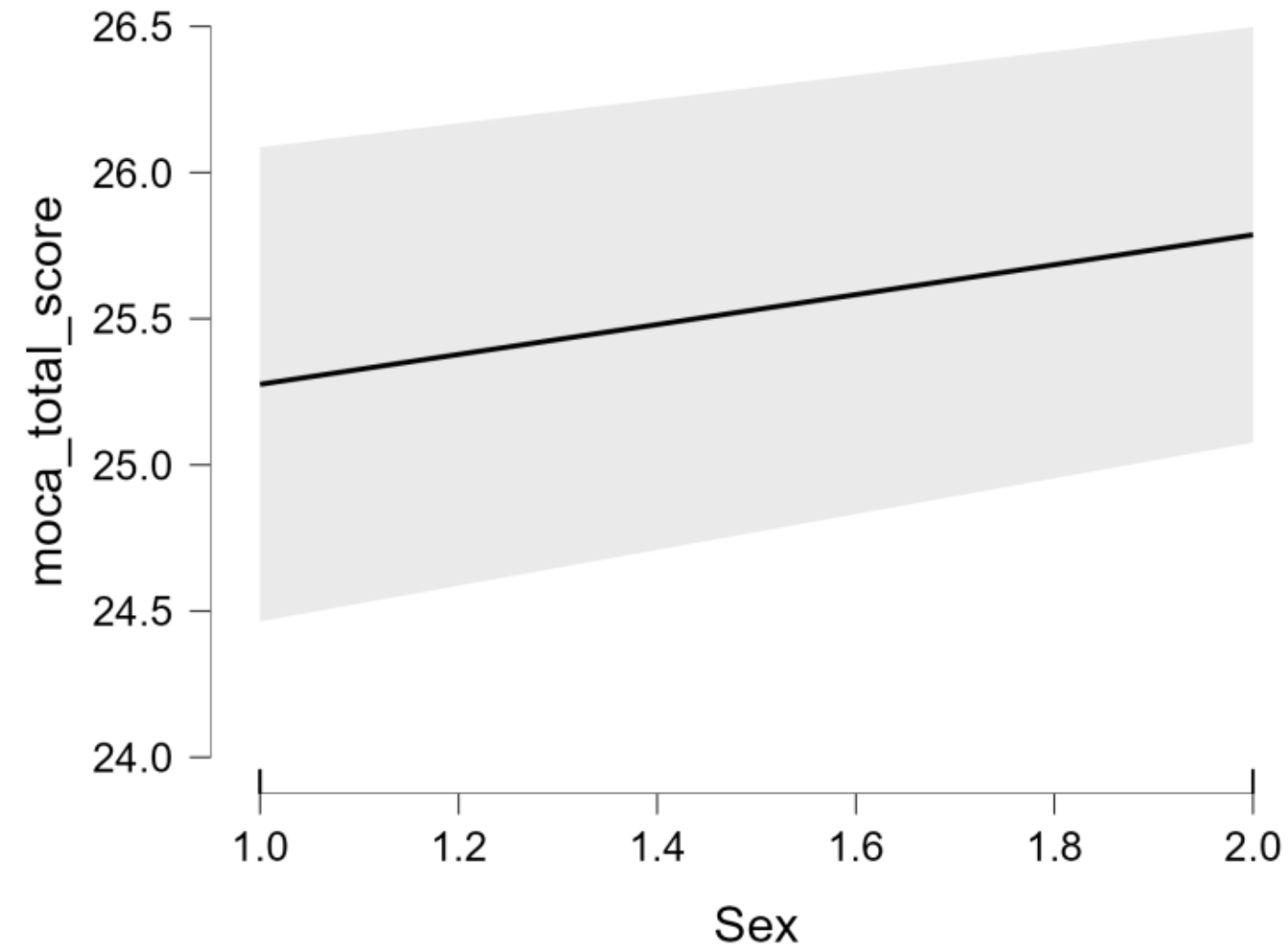

Marginal effect of Age on moca_total_score

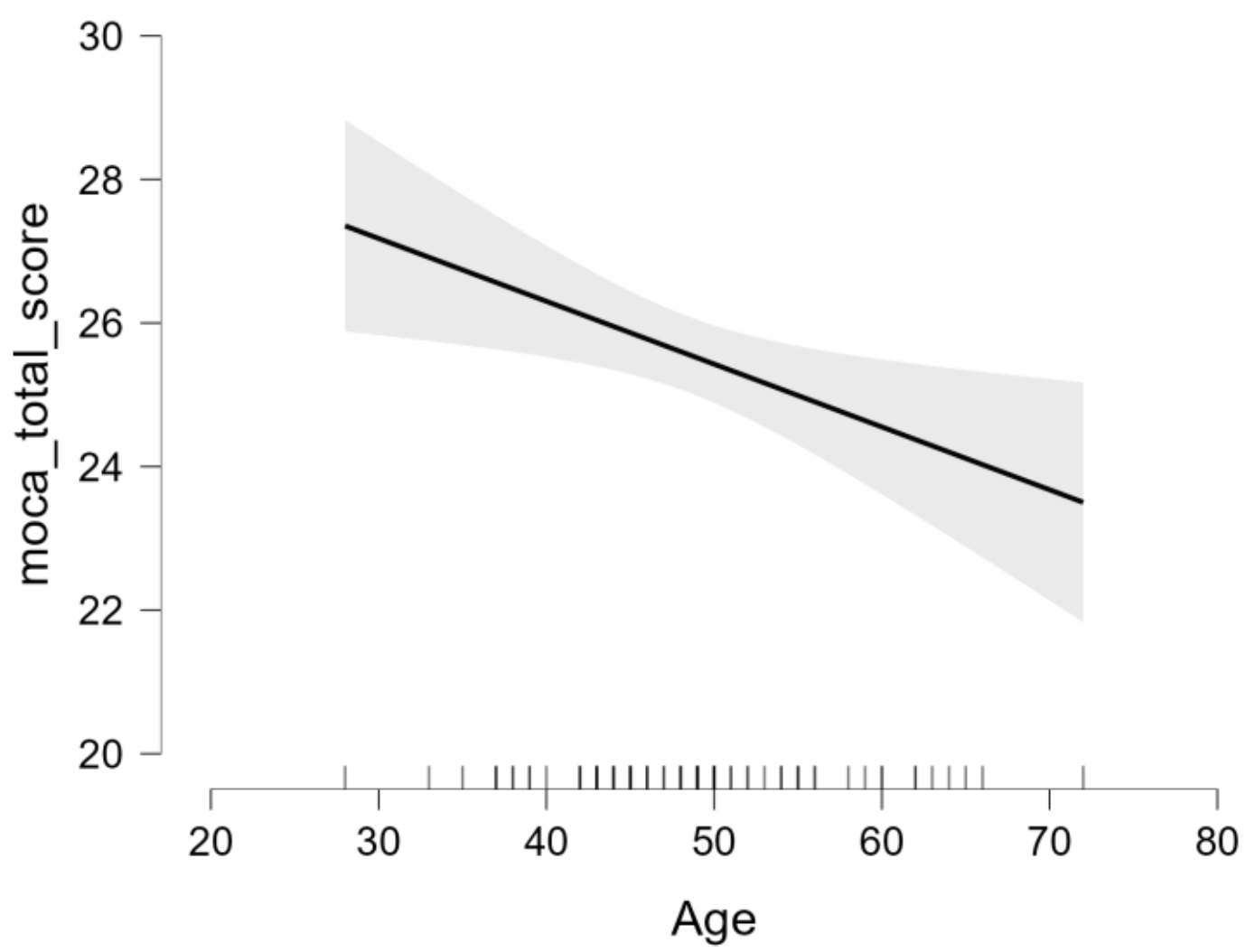

Supplement: Supplementary file 4 [file SJOP-67-1359-s005.pdf]
